# New diagnostics for tuberculosis: fulfilling patient needs first

**DOI:** 10.1186/1758-2652-13-40

**Published:** 2010-10-25

**Authors:** Jean-François Lemaire, Martina Casenghi

**Affiliations:** 1 Médecins Sans Frontières Campaign for Access to Essential Medicines, Geneva, Switzerland

## Abstract

**Background:**

An effective tuberculosis (TB) control programme requires early diagnosis and immediate initiation of treatment. Any delays in diagnosing TB not only impair a patient's prognosis, but also increase the risks of transmitting the disease within the community. Unfortunately, the most recent TB diagnostic tools still depend on high-infrastructure laboratories, making them poorly adapted for use in resource-limited settings. Additionally, existing tests show poor performance in diagnosing TB in children, people living with HIV/AIDS, and extrapulmonary forms of the disease. As a consequence, TB patients are still to date left with either *fair *access to *poor *diagnostics or *poor *access to *fair *diagnostics.

**Discussion:**

This article discusses recent efforts to identify the minimal test specifications for a new TB point-of-care diagnostic test through an approach based on medical and patient needs. As a first step, survey interviews with field practitioners were designed in order to identify the top-priority medical needs in resource-limited settings concerning new TB diagnostics. Subsequently, an expert meeting convening field practitioners, laboratory experts, diagnostic test developers and researchers was held with the objective of defining the minimal test specifications for a new TB point-of-care test that would meet the identified medical needs. Finally, gaps in, as well as potential solutions for, enabling the development of adequate, patient needs-driven, low-cost new TB diagnostic tests specifically designed for vulnerable populations are discussed.

**Summary:**

Any new TB point-of-care diagnostic test should be designed to meet minimal specifications satisfying the most urgent medical needs in resource-poor settings. The major gaps for developing a new TB point-of-care test include identification of new biomarkers, simplification of technological platforms, development of adequate and accessible specimen banks, and identification and definition of reference standards for diagnosis of childhood TB. Innovative research and development funding ensuring de-linkage of research and development costs from the price of the new product, such as a prize fund mechanism, could help focus these efforts towards the delivery of a much-needed point-of-care diagnostic test for TB.

## Background

Tuberculosis (TB) is a major public health problem associated with more than 9.4 million incident cases and almost 1.8 million deaths in 2008 alone: this is the equivalent of 5000 people dying every day [1]. TB remains the world's largest treatable infectious cause of death, with 90% of patients living in resource-limited settings [2], and the African continent having 14 of the 15 highest-burden countries in the world [1]. More importantly, an estimated 60% of patients seeking care are found at health-post level or peripheral health clinics, where adequate laboratory infrastructure to perform TB laboratory investigations often do not exist, not even through sputum smear microscopy (SSM) [3]. Thus, the need to adapt the diagnostic tools to the burden and reality of the epidemic is crucial.

Although the ideal characteristics for the design of a diagnostic test for resource-limited settings have been suggested as Affordable, Sensitive, Specific, User-friendly, Rapid, Equipment-free, and Delivered (the ASSURED system) to those in need [3], all existing methods and those under development do not fulfil many of these criteria. Because of its low cost, long history and basic laboratory infrastructure needs, SSM remains the most widely used TB diagnostic test. However, the low sensitivity of the SSM method itself (<60% in immunocompetent patients) [4] emphasizes the need for a new TB diagnostic test.

To meet the pressing needs for a point-of-care (POC) test, defined here as a test that can be performed at least (but not exclusively) at remote health care-structure level (e.g., rural health posts or mobile clinics), several immunochromatographic assays, so-called lateral flow devices or rapid diagnostic tests [5], have been developed and commercialized. However, performance data of such TB assays have consistently shown poor clinical relevance [6]. Notably, a recent evaluation by the World Health Organization (WHO) Special Programme for Research and Training in Tropical Diseases showed that of 19 rapid diagnostic tests studied, all performed insufficiently and were inadequate for recommendation in TB diagnosis algorithms [7].

Although in 2006, potential public health gains from a new TB diagnostic test were reported to likely rise proportionally with increased access to testing [8], the most recent TB diagnostic tools still continue to depend on high-infrastructure laboratories. Recent research efforts have led to either the development of new tools or the improvement of existing methods [9]. Although some of the recent microscopy improvements offer true advantages over the conventional method, their overall detection yields remain poor [5,10]. Other methods, such as mycobacterial liquid culture, have helped improve detection yields and reduce delays. However, their time to result, as well as high infrastructure and training needs, substantially hinder their use, particularly in resource-limited settings. Recent years have also seen progresses in the diagnosis of multidrug-resistant TB (MDR-TB), thanks to the development of nucleic acid amplification-based tests.

Of particular interest for implementation in resource-limited settings are line probe assays, the implementation of which was recommended by WHO in 2008 [11], and the recently marketed Xpert MTB/RIF [12]. While line probe assays have helped accelerate diagnosis of drug resistance, their use is limited to sputum smear-positive patients, and their implementation is only possible in high-level infrastructure laboratories. The Xpert MTB/RIF certainly represents an interesting advance with data from evaluation studies showing promises of high detection yield for TB and resistance to rifampicin in smear-positive, as well as smear-negative, patients. The Xpert MTB/RIF also has the potential to be used in moderately equipped laboratories. However, this device does not fulfil the need for a POC diagnostic test that can be implemented in the most peripheral settings, e.g., rural health centres, which often have highly limited infrastructure and resources and are not suited for operating and maintaining real-time polymerase chain reaction (PCR)-based equipment with that design.

Although TB care is still delivered at central health facilities in many settings, efforts aimed at decentralizing TB and MDR-TB treatment are showing success in shortening time to initiate treatment and improving treatment outcomes [13-17], suggesting that delivery of care at community level can represent an effective strategy to improve TB control. However, the impact of a decentralized model of care is limited by the lack of laboratory-free TB diagnostics suitable for field use and the need to rely on referral to central facilities for proper TB diagnosis. Concomitant strengthening of central laboratories certainly must be planned for performance of confirmatory tests and drug susceptibility testing (DST). However, this should be done in parallel with decentralizing TB diagnosis and treatment in order to improve access to care.

The type of specimen required by diagnostic tests also represents a challenge for TB diagnosis. All routine laboratory-based TB diagnostic methods available to date depend on respiratory specimens. Such specimens are highly susceptible to significant quality variability and therefore have limited diagnostic utility for some patient populations. Paradoxically, the two most vulnerable populations to TB infection, infants and people living with HIV/AIDS, are either unable to produce sputum specimens or are likely to produce paucibacillary specimens, respectively. As a result, these patient populations can only have access, when available, to diagnostics of suboptimal performance.

Although we appreciate the strong efforts that have been made in the current pipeline of product development [18], the most advanced new tools will still either require high-level infrastructure needs or will offer only a limited increase in performance. Other methods are currently in early phases of development, such as loop-mediated isothermal amplification [19], MPT64 skin patch [20], transrenal urinary DNA detection [21], antibodies in lymphocyte supernatant assay [22], and beta-lactamase enzymatic assays [23]. These technologies should be adapted to a POC platform whenever possible.

Considering constant advances in miniaturization technologies, applied sciences and engineering, new possibilities for the development of a TB POC test could exist in the near future. It is imperative that any new TB test provide new assets to the current TB diagnostics environment by adequately fulfilling the medical needs and field-operational limitations faced by TB practitioners in the most endemic regions.

## Discussion

### Expert survey: keeping an ear to the ground

With the objective of identifying, discussing and answering key medical questions about the development of a new test for TB, Médecins Sans Frontières, the Treatment Action Group and Partners In Health designed a questionnaire [24] ("Expert Opinion Check") targeting TB field practitioners. A total of 30 survey respondents were reached, including field clinicians (n = 21; three paediatricians; two were also laboratory experts) and laboratory specialists (n = 9) from 17 medium- and high-burden countries (five from Asia, 10 from Africa, one from eastern Europe and one from Latin America). These professionals were affiliated with TB programmes operated and/or supported by different types of organizations and institutions (national TB programmes, n = 13; academic institutions, n = 2; non-governmental organizations, n = 16). Survey participants represented a heterogeneous group of professionals involved at all levels of care, from hospitals to rural health posts, and also included specialists in charge of national TB programmes or working in research institutions.

The Expert Opinion Check survey was conducted from 30 January to 24 February 2009. Data were captured by telephone interviews, and the survey was composed of 21 open, semi-open and ranking questions, covering: (1) the context of TB practice of the participant; (2) shortcomings of current diagnostic tools; (3) intended use of a new TB POC test; (4) targeted patient population(s) of a new POC test; and (5) desired specimen sample type(s). The survey therefore focused on the major gaps currently seen in TB diagnosis and on the intended use for a new TB POC test.

To identify the major barriers currently seen in TB diagnosis, participants were asked to identify the five highest priority gaps needing to be addressed. The inadequacy of sputum as a specimen sample in diagnosing paediatric TB, HIV/TB co-infected patients, extrapulmonary TB (EPTB) patients and low sensitivity of SSM emerged as the biggest limitations in TB diagnosis. This was followed by lack of drug susceptibility evidence without further referral, low overall diagnostic performance of SSM due to variability of analysis, and lengthy turnaround time to obtaining results of current tests.

Consistent with this, when participants were asked to identify additional patient populations who should be diagnosed by a new TB POC test, HIV/TB co-infected patients emerged by far as the highest priority, followed closely by paediatric suspected cases. Smear-negative patients, drug-resistant TB patients and EPTB patients were also indicated as important populations. Patients affected by latent TB and patients at risk of dying quickly were not perceived as priority populations whose diagnosis should be targeted with a new test.

Finally, in order to understand what test characteristics are most important from the end user's perspective, participants were asked to choose what test they would prefer among a range of tests varying in sensitivity and ability to detect TB in different patient populations. One extreme of the range was represented by a test characterized by high sensitivity (90%) and specificity (95%), but with the ability to detect pulmonary TB only in HIV-negative adults. The other extreme was represented by a test with sensitivity and specificity comparable to SSM (60% and 95%, respectively), but with the ability to detect TB and provide DST in all patients, irrespective of age and HIV status. The vast majority of participants chose a test with sensitivity of 75% and specificity of 95%, but with the ability to diagnose TB in all patients, irrespective of age and HIV status. Thus, surveyed participants traded off test sensitivity to a certain extent in favour of the ability to detect TB in a broader population. However, they would not be satisfied with a test having the same poor sensitivity performance as that currently seen with SSM, even if such a test would be able to detect TB in a broader population.

To summarize, the survey respondents generally desired a new TB POC test that, in addition to increased sensitivity compared with SSM, can, as a minimum: diagnose active pulmonary TB in all patients (independent of age or HIV status) within a day; support a treatment initiation decision; be easy to use by nurses or community health workers; use capillary blood, urine or breath samples; and preferably provide DST information. The main survey findings are listed in Table 1. The complete survey analysis report is freely accessible online [25].

**Table 1:** Main preference trends from the Expert Opinion Check survey for the requirements of a new TB POC test

**Intended use**	To diagnose active pulmonary TB
**Medical decision to be influenced**	Treatment initiation
**Populations targeted**	All, including infants and HIV co-infected
**Test user**	Nurses or community health workers
**Level of healthcare structure**	Closest to where patients can be treated
**Sample types**	Capillary blood, urine, or breath
**Time to results**	<1 day
**Confidence level of results**	>75%
**Optional, but highly needed**	Drug sensitivity testing information

### Expert meeting: finding common ground in defining minimal test specifications

The detailed outcomes from the survey analysis were presented during a two-day meeting, entitled "Defining Specifications for a TB Point-of-Care Test", held in Paris, France, in March 2009, with the main objective of discussing and reaching consensus on the minimum technical specifications for a POC TB diagnostic test that meets medical needs in resource-limited settings.

The meeting had three defined objectives: (1) to reach a consensus on priority medical needs that should be fulfilled by a new TB diagnostic test; (2) to reach a consensus on the minimum POC test specifications required to meet those medical needs and that are technologically feasible in a five- to 10-year timeframe; and (3) to analyze the most promising research and development (R&D) pathways that can lead to the delivery of such a test in a five- to 10-year timeframe.

The meeting group was composed of 34 participants with recognized expertise in a wide range of relevant areas, including clinicians and laboratory experts with significant field experience in resource-limited settings (additional to the survey respondents), representatives from patient community groups, test developers, and research scientists working in the area of TB diagnostics. Such a multidisciplinary group was brought together with the aim of enabling a fruitful, cross-disciplinary dialogue between end users and test developers and to ensure the translation of medical needs into test specifications that would be feasible on the basis of the technological and scientific advances. This meeting was conceived to be a first step in a process of defining specifications for a new TB POC test driven by medical needs in resource-limited settings.

Further discussions among the group members led to an overall consensus on the relevance of the top-priority medical needs previously identified through the survey (Table 1). Particularly, the group agreed that the highest priorities were having a TB diagnostic test adapted to resource-limited settings in a portable POC format, as well as adapted for all patient populations, including infants and individuals co-infected with HIV.

As to the second objective of the meeting, the group also achieved a consensus on the specifications that a new TB POC test should minimally meet in order to fulfil the most urgent needs. Table 2 illustrates the general key minimal specification criteria agreed upon. Indeed, through a prioritization exercise, the group identified the essential test specification characteristics for any new TB POC test. These "untradeable" test specification features were sensitivity, specificity, rapid test performance/time to results, simple sample preparation and an unambiguous readout.

**Table 2:** Minimum test specifications identified during the March 2009 experts' meeting, "Defining Specifications for a TB Point-of-Care Test"

Criteria	Minimum specifications required
Medical decision	Treatment initiation
Sensitivity, adults (regardless of HIV status)	Pulmonary TB: Smear positive, culture positive: 95% Smear negative, culture positive: 60-80% (no agreement on a minimum) (detection of extrapulmonary TB preferred, but not required)
Sensitivity, children (regardless of HIV status)	80% compared to culture of any specimen and 60% of probable TB (noting the lack of a gold standard)
Sensitivity, extrapulmonary TB (regardless of HIV status)	80% compared to culture of any specimen and 60% of probable TB (noting the lack of a gold standard)
Specificity	Adults: 95% compared with culture Children: 95% compared with culture, 90% for culture negative probable TB (noting the lack of a gold standard)
Time to results	Maximum 3 hours (patient must obtain same-day results, desirable would be <15 minutes)

Note: The group could not reach consensus on: (1) the minimal sensitivity in smear-negative adults; (2) the diagnosis of extrapulmonary TB in adults as a minimal requirement; and (3) the rejection of use of sputum as a sample.

The meeting also included in-depth discussions on whether to include certain specific criteria as absolute minimal requirements, notably the minimal sensitivity in smear-negative adults, diagnosis of EPTB in adults, and rejection of use of sputum as a specimen type. Since no definite agreement could be reached on these three specific criteria, an interim decision was made by all participants that these criteria should be considered as highly desirable, but not included as minimal requirements. For details, the complete meeting report is freely available online [26].

In analyzing the most promising R&D pathways that can lead to the delivery of such a test, the meeting group met the third objective and identified the following four major gaps that need to be urgently filled to facilitate the development of a new POC TB test within five to 10 years:

#### (i) Identify new biomarkers to use with existing POC platforms

Bridging this gap requires the performance of proof-of-principle validation screening of potential biomarkers (antigens and/or antibodies) in a standardized way, as well as standardized evaluation of combinations of earlier-verified biomarker candidates. These two steps are critical to allow for fast-tracked POC test development using existing rapid immunodiagnostic test platforms, namely lateral flow assay devices (dipsticks). To date, no biomarker tested on lateral flow devices has shown sufficient performance for diagnosing active TB [5,6]. However, the expert group recognized that combinations of potential biomarkers need to be explored further as they could provide better yield in terms of sensitivity and specificity.

#### (ii) Develop a new POC platform for existing DNA/molecular biomarkers

Considering that molecular regions of mycobacterial DNA have been identified for the detection of TB from clinical specimens, major scale-up efforts are needed to simplify and accelerate the engineering of diagnostic platform technologies for DNA amplification and detection in a portable, field-adapted POC device. Although the Xpert MTB/RIF is not suitable for implementation in its current design in resource-limited, peripheral settings, it represents an interesting step forward in terms of simplification of a PCR-based test and development of a closed-system technology less prone to contamination. The development of the Xpert MTB/RIF test should encourage exploring possibilities for further simplification of similar technologies. Moreover, DNA detection seems to show high performance similar to culture and could allow for the use of alternative specimen types (e.g., urine, stool).

#### (iii) Specimen banks

During early R&D phases of a new diagnostic test, researchers must have access to clinical samples from specimen banks to validate the proof-of-principle of candidate biomarkers and new method prototypes in their laboratories, as well as to subsequently evaluate new test prototypes. Academic researchers and test developers at the meeting clearly highlighted the need for specimen banks to include a wide variety of specimen types, including specimens from HIV co-infected patients and individuals of all ages, particularly children. Although it is recognized that specimen banks themselves will not drive the development of a new test, there is an increasing consensus that specimen banks are an important tool enabling and facilitating the development process [27]. The group also recommended that the adequacy and accessibility of existing specimen banks should be assessed. If the quality standards or accessibility of existing specimen banks are found to be unsatisfactory and cannot be improved, a reliable, open-access specimen bank should be created. An assessment of the adequacy and accessibility of existing banks is ongoing, and access to this information will be made public.

#### (iv) Funding

According to estimates from the Treatment Action Group, trends for 2005 to 2007 showed that TB R&D funding experienced an alarming shortfall [28]. In 2007, the last year analyzed in the report, the total amount invested in TB R&D was $482 million. Considering that this amount covers multiple research investment categories, what was left specifically for diagnostics was around $42 million (8.7%). The meeting participants identified this amount as being insufficient to cover the R&D needs in TB diagnostics, and estimated that current investment for TB diagnostics R&D needs to be increased at least four-fold. Additionally, the group highlighted the need for new financing mechanisms, such as a prize fund (see next section), that could concentrate the efforts of researchers and test developers towards new innovations leading to the creation of a new TB POC test.

Outside of these four major gaps, the group also identified as a high priority the need to overcome the lack of an accurate clinical TB case definition for children. Indeed, this problem was identified as a major hurdle in the validation of new diagnostics suitable for children and will require the establishment of a proxy infant gold standard.

### Hitting the ground running in stimulating new innovations

The 2010 World TB Day theme, "On the Move against Tuberculosis, Innovate to Accelerate Action", is appropriate. To spur innovation for a new TB POC test, not only more funds, but also new ways of allocation, will be needed. The World Health Assembly, through its global strategy and plan of action on public health, innovation and intellectual property, adopted a clear framework for action to explore ways to foster innovation, build capacity and improve access to health products in developing countries. The process led to agreed-upon recommendations to investigate new mechanisms, such as public-private partnerships, patent pools, advanced purchase commitments and prize funds, to ensure the creation of affordable diagnostics adapted to resource-constrained settings [29-31].

One of these innovative financing initiatives is the prize fund mechanism, which has been proposed as an alternative to patents and product monopolies, and designed to reward R&D innovation while ensuring access to the final products. Unlike the current patent system, a prize immediately serves as the compensation for R&D investment, negating the need to recoup this investment through high end-product prices ("de-linkage"). If designed appropriately, a prize competition would also serve to direct R&D towards specifically identified needs, since it would set specifications that successful developers would need to meet.

The governments of Bangladesh, Barbados, Bolivia and Suriname submitted several R&D financing proposals based on prize funds to the WHO Expert Working Group on R&D financing at its first public hearing in April 2009 [32]. This included a proposal for a $100 million prize fund strategy overseen by the WHO for a new, low-cost, rapid TB POC test that would assume the fixed cost of clinical trials [33].

To minimize barriers to entry, all potential competitors, especially competitors in developing countries, must have access to sufficient starting funds. Strikingly, the Bill & Melinda Gates Foundation, through its Grand Challenges in Global Health Initiative, has recently announced that it will make $30 million available for the first phase of its POC Diagnostics Grant Opportunity [34].

## Summary

While the survey opinions of practitioners in resource-limited settings reflected patient medical needs, experts from a multidisciplinary group agreed that any new TB POC test should minimally achieve specifications that meet those medical needs. To reach this ultimate objective, efforts should be made to address the four major gaps identified, namely, the identification of new biomarkers, development of new POC technological platforms, establishment of adequate specimen banks, and increased funding dedicated to TB diagnostics R&D. Additionally, a reference standard for evaluation of TB diagnostics in children should be identified.

Alternative financing mechanisms should be established in order to foster new innovations in a way that delinks the cost of R&D and the price of the end product. Some of the proposed mechanisms could potentially allow more idealistic objectives and therefore lead to a new TB POC test that fulfils field-based medical needs.

Policymakers and funding agencies should act with urgency and prioritise funding tracks enabling the development of a new TB POC diagnostic test suitable for all people in need, including infants and individuals co-infected with HIV/AIDS, ideally based on non-invasive, non-respiratory clinical specimens and able to give DST information. Failure to address this massive need will continue to result in the unnecessary deaths of almost 2 million individuals from TB every year.

## Competing interests

The authors declare that they have no competing interests.

## Authors' contributions

J-FL led the design and analysis of the Expert Opinion Check experts' survey, developed the scientific content of the experts' meeting, and drafted the manuscript. MC contributed to the development of the experts' meeting agenda and analysis of meeting outcomes. All authors read and approved the final manuscript.
